# Nutrition education has a positive impact on nutritional knowledge, food consumption and body composition in PLHIV: a randomized clinical trial

**DOI:** 10.3389/fpubh.2026.1725861

**Published:** 2026-05-20

**Authors:** E. Alana D. Fernandes, Phelipe Wilde, Paulo F. de Almeida-Neto, Júlio César Medeiros Alves, Allana Rebeca da Silva Rogas, Lavínia Beatriz Fernandes Souza, Breno Guilherme de Araújo Tinôco Cabral, Paulo Moreira Silva Dantas

**Affiliations:** 1 Health Sciences Center, Federal University of Rio Grande do Norte, Natal, RN, Brazil; 2 Nutrition Department, Federal University of Rio Grande do Norte, Natal, RN, Brazil; 3 Physical Education Department, Federal University of Rio Grande do Norte, Natal, RN, Brazil

**Keywords:** adequate dietary, food and nutrition education, HIV infections, nutritional assessment, public health

## Abstract

Antiretroviral therapy (ART) has substantially increased the life expectancy of people living with HIV (PLHIV), but its prolonged use has been associated with metabolic alterations that may negatively affect nutritional status. In this context, knowledge about food and nutrition plays a crucial role in promoting healthy food choices and preventing health complications. This study aimed to assess the impact of an intervention with Nutrition education (NE) on nutritional knowledge, food consumption, energy expenditure and body composition in PLHIV. A randomized, controlled, unblinded clinical trial was conducted16 participants of both sexes. The results showed an interaction effect between time (baseline, 30 days and 60 days) and condition (intervention vs. control), with significant differences in nutritional knowledge: experimental intra-group (*p* = 0.015) and between groups (*p* = 0.005), both at 60 days; in food consumption between the groups (*p* = 0.014) and within the control group (*p* < 0.001), both at 60 days; in body fat percentage within the experimental group at 30 days (*p* = 0.070) and 60 days (p = 0.001) and; in body mass within the experimental group at 60 days (*p* = 0.034). *Post hoc* analyses demonstrated significant between-group differencesin nutritional knowledge (*p* < 0.001), food consumption (*p* = 0.002), body mass (*p* < 0.001) and total body fat (*p* = 0.016). The NE intervention was effective in increasing nutritional knowledge, improving the frequency of consumption of foods with higuer nutritional density and reducing body mass and body fat among PLHIV after 60 days.

## 1 Introduction

The introduction of high-potency antiretroviral therapy (ART) in the early 1990s substantially increased the life expectancy of individuals living with human immunodeficiency virus (HIV) by slowing disease progression (1, 2). Following the widespread adoption of ART, HIV-related mortality declined markedly, accompanied by significant improvements in quality of life. However, accumulating evidence indicates that long-term ART use is associated with complex metabolic alterations that adversely affect nutritional status, energy expenditure, glucose metabolism, and lipid profile regulation (3–6).

Recent studies assessing the nutritional status of people living with HIV (PLHIV) have reported a high prevalence of overweight and obesity, alongside a low prevalence of undernutrition (7, 8). A study conducted in the United States demonstrated a significant increase in the proportion of overweight and obese individuals following the introduction of ART (9).

Regarding dietary intake, another study reported a higher consumption of foods rich in cholesterol, trans and saturated fats, simple carbohydrates, and sodium, along with a low intake of roots and tubers and fiber-rich foods, such as oats and fruits (7).

Nutrition education (NE) has demonstrated positive effects on improving the quality of life and overall well-being of people living with HIV (PLHIV) (10). In official resolutions, the World Health Organization (WHO) and the Academy of Nutrition and Dietetics have emphasized the importance of nutrition and dietetics professionals worldwide providing appropriate NE guidance for PLHIV, with the goal of improving health outcomes given the chronic nature of HIV infection and the metabolic consequences of antiretroviral therapy (8, 11).

Nevertheless, limited access to nutritional care within public health services hampers the provision of nutritional therapy and may contribute to clinical deterioration among PLHIV. Increasing awareness of healthy eating promotes changes in dietary behavior; therefore, food and nutrition knowledge may be directly associated with healthier food choices and improved adherence to HIV treatment (12, 13).

In this context, the present study hypothesizes that NE can positively contribute to improvements in food consumption, body composition, and resting energy expenditure through the acquisition of nutritional knowledge. Accordingly, the aim of this study was to evaluate the impact of NE practices on these nutritional parameters, with an emphasis on promoting food autonomy and empowering PLHIV to make healthier dietary choices.

## 2 Methodology

### 2.1 Study design

This study was designed as a randomized controlled clinical trial without blinding. Randomization was performed using the open-source software Jamovi^®^ (Version 2.3.18, Sydney, Australia). The allocation sequence was concealed from recruiters and was accessible only to the registered dietitian responsible for delivering the intervention. Neither the participants nor the principal investigator, who was the nutrition professional implementing the intervention, were blinded to group assignment.

To minimize the risk of bias, participant recruitment and data collection were conducted by a team of laboratory technicians independent of the research team. Statistical analyses were performed by an external collaborator who had no involvement in data collection or intervention delivery. The study was reported in accordance with the Consolidated Standards of Reporting Trials (CONSORT) checklist (14).

Twenty adults living with HIV were recruited from a university extension program aimed at PLHIV, that provides guidance on physical exercise and nutritional counseling to promote health, quality of life and wellbeing. The program is conducted at the Federal University of Rio Grande do Norte, located in the city of Natal, Rio Grande do Norte, Brazil. However, to prevent the groups from interacting within the same program environment, each group accessed the project space at different times.

Participants were randomily allocated into two groups, an intervention group and a control group. Both groups underwent all the assessments at baseline, 30 days and 60 days however only the intervention group received the NE intervention and nutritional monitoring.

**Figure 1:**
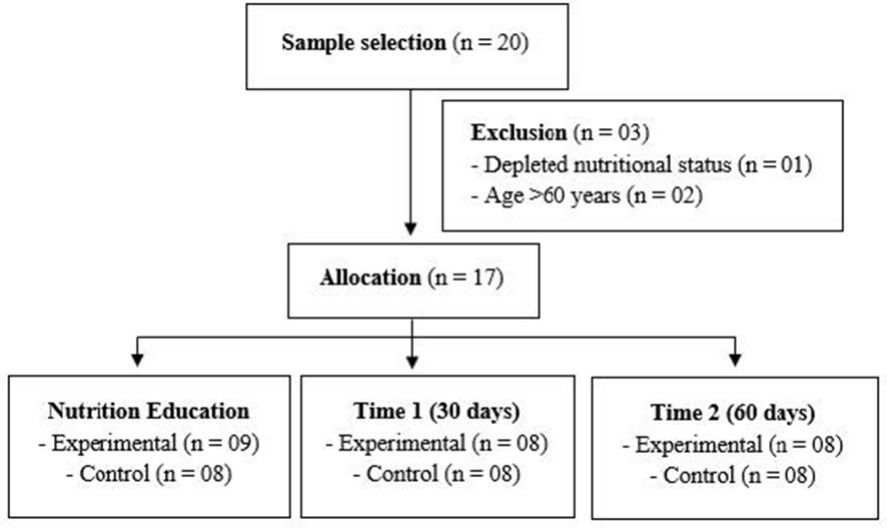
Study flowchart. Source: own authorship.

Eligible participants were adult men and women living with HIV who had been receiving ART for at least six months and who were physically active, defined as engaging in physical activity on at least five days per week and accumulating a minimum of 150 minutes of physical activity per week. The exclusion criteria were: non-compliance with the recommendations for carrying out the assessments, non-attendance at all stages of the study and depleted nutritional status, based on the Body Mass Index (BMI) classification (< 18.5 kg/m^2^ - underweight) (Figure 1) (15).

An *a priori* sample size calculation was performed considering a small effect size of 0.30, an alpha level of 0.05, and a statistical power (beta) of 0.80. The design included two groups (control and experimental) and four variables: body fat, basal energy expenditure, dietary intake, and nutritional knowledge.

Sample size estimation was conducted using G^*^ Power (version 3.1, Düsseldorf, Germany), applying the F statistic for repeated measures (baseline, 30 days and 60 days). The analysis indicated a minimum sample size of 16 participants (50% allocated to each group), corresponding to a statistical power of 0.84 (F-critical = 2.5). To account for potential attrition, we recruited 25% more participants than the minimum required sample size, resulting in a total of 20 participants (50% allocated to each group).

**Figure 2:**
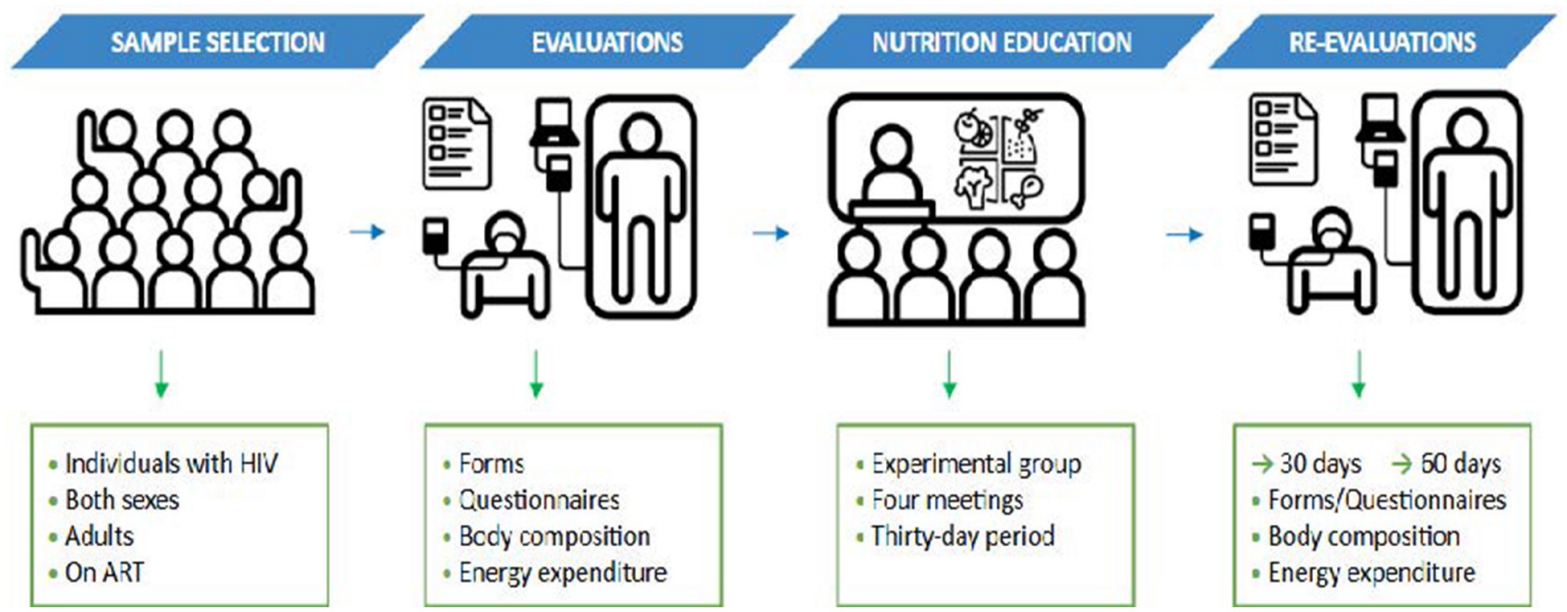
Study design. Source: own authorship.

In the first phase of the study, volunteers were recruited and selected. The second phase involved baseline assessments, including completion of the characterization form, body composition evaluation, assessment of resting substrate oxidation, and completion of nutritional knowledge and dietary intake questionnaires. The third phase consisted of the NE. The fourth phase involved follow-up assessments (of body composition, resting energy expenditure, nutritional knowledge, and dietary intake) at 30 and 60 days after the intervention (Figure 2).

Participants who agreed to take part in the study completed a form containing sociodemographic information and subsequently underwent anthropometric assessment. BMI, defined as weight in kilograms divided by height in meters squared, was used as the anthropometric indicator, with classification based on the cutoff points established by the WHO (16).

Body mass was measured using an electronic scale (MIC-RS232, Micheletti^®^, São Paulo, Brazil) with a capacity of 500 kg, while height was assessed using a portable floor stadiometer (Est-223, Balmak^®^, São Paulo, Brazil) (15). Waist circumference was measured with the participant in a standing position, at the midpoint between the last rib and the iliac crest, using a flexible, non-elastic fiberglass anthropometric tape with a scale from 0 to 150 cm and a resolution of 0.1 cm (Sanny^®^, São Paulo, Brazil). This procedure was repeated three times to obtain the average waist circumference, which was classified according to pre-established cutoff points for men and women (17).

Physical activity level was assessed using the short version of the International Physical Activity Questionnaire (IPAQ), which has been validated for the Brazilian population (18). Data were analyzed according to frequency and duration criteria established by the consensus between CELAFISCS and the Centers for Disease Control and Prevention (CDC) in Atlanta in 2002 (19), which proposes classification into five categories: very active, active, irregularly active and sedentary.

### 2.2 Ethics

The study was approved by the Research Ethics Committee of the Onofre Lopes University Hospital at the Federal University of Rio Grande do Norte (CAAE: 75829023.2.0000.5292/opinion #6.573.188). After being informed about the study's objectives, procedures, and potential benefits, individuals who agreed to participate signed an Informed Consent Form.

The study was registered in the Brazilian Clinical Trials Registry (https://ensaiosclinicos.gov.br/rg/RBR-57vfs7m) under the Universal Trial Number (UTN) U1111-1304-8090, with a registration date of November 6, 2024.

### 2.3 Assessment of dietary intake markers

Dietary intake was assessed using the food consumption markers questionnaire proposed by the Food and Nutrition Surveillance System (SISVAN) (20). The questionnaire consists of ten items addressing the usual frequency of consumption of ten foods/food groups over the past 7 days. The version used in this study was the previous version of the instrument, as it better suited the research objectives.

This is a simple and objective tool that enables a qualitative assessment of dietary intake by investigating the frequency of consumption of unprocessed and minimally processed foods (items 1–5), as well as processed and ultra-processed foods (items 6–10). This allows for an analysis of adequate or inadequate consumption of each item.

According to the Brazilian Dietary Guidelines, consumption of unprocessed and minimally processed foods was considered adequate when reported ≥5 times per week, while consumption of processed and ultra-processed foods was considered inadequate when reported >2 times per week (21).

### 2.4 Assessment of nutrition knowledge

The assessment of nutrition knowledge was conducted using the “Nutrition Knowledge Questionnaire for Adults” (NKQA) (22), adapted for the purposes of this study. Questions 5, 17, and 20 were removed, as they were not addressed during the intervention, resulting in a total of 17 questions distributed according to the following content areas: Group 1 – Diet and associated diseases (questions 1, 2, 3, and 4); Group 2 – Fruits, vegetables, and fiber (questions 5, 6, and 7); Group 3 – Fats, sugars, and salt (questions 8, 9, 10, 11, 12, and 13); Group 4 – Healthy food choices (questions 14 and 15); and Group 5 – Nutrition labeling (questions 16 and 17).

Scoring was calculated based on the criteria established by the researchers: partially correct = 0.5 point; correct = 1 point; and incorrect = 0 points, totaling a maximum of 31 points when considering each item of every question. Thus, higher scores indicated greater nutrition knowledge (22).

### 2.5 Assessment of oxidation of energy substrates at rest

Participants were instructed to sleep approximately 8 h the night before, to fast for 12 h, and to refrain from engaging in physical activity and consuming caffeine- or alcohol-containing beverages for 48 h prior to testing. These procedures aimed to minimize the influence of the thermic effect of food and physical activity on resting metabolism.

Indirect calorimetry was performed using a Respiratory Gas Analyzer (Cortex Metalyzer 3B, Biophysik, Leipzig, Germany). Volunteers remained in a supine position on a mattress on the floor, wearing a face mask for 10 min with the device disconnected, followed by an additional 20 min with the device connected to assess substrate oxidation at rest. Measurements were conducted under conditions of dim lighting, absence of noise, ambient temperature controlled at 24 °C, and relative humidity around 50%.

From the energy expenditure data, the first 3 min of measurement were excluded to account for participant adaptation to the equipment, which commonly causes initial fluctuations. The mean of the remaining values was used to estimate daily resting energy expenditure. Based on oxygen consumption (VO_2_) and carbon dioxide production (VCO_2_), the respiratory quotient (RQ) was calculated, and gas exchange data were used to quantify daily energy expenditure in kilocalories (kcal/day).

### 2.6 Body composition assessment

The participants body composition was examined using dual-energy X-ray absorptiometry (DXA) (GE^®^, Prodigy Advance model, GE Lunar software [version 15.0.0], Madison, USA). The detector captures information associated with the attenuation of photon beams after passing through the body of the person being evaluated and sends it for analysis on a microcomputer using specific software. The DXA equipment was configured as follows for whole-body assessment: voltage (kV) 76.0, current (mA) 0.150, and radiation dose (μGy) 0.4, which is classified as a very low dose with no associated health risks.

The data used in the study were body mass in kilograms (kg), BMI, total body fat in kg, total body fat percentage, fat-free mass (FFM), and the identification of the android/gynoid fat ratio (A/G ratio).

Gynoid fat is predominantly located in the pelvis and thighs, presenting a relatively low cardiovascular risk. In contrast, android, or central/trunk, fat is more centered in the abdominal region and is associated with a higher risk of metabolic complications. Thus, an android to gynoid fat ratio greater than 1 (indicating a predominance of android fat) is associated with an increased risk of metabolic changes (23).

Body fat assessment was performed according to the standards established by Pollock and Wilmore, considering the following average percentages according to gender and age group: males 18%−20% (18–35 years), 21%−23% (26–45 years), and 24%−25% (46–65 years); female 24%−25% (26–35 years), 27%−29% (36–45 years), 29%−31% (46–55 years) and 30%−32% (56–65 years) (24).

### 2.7 Nutrition education intervention

NE was delivered by a registered nutritionist holding a bachelor's degree in Nutrition and a specialization in Nutrition and Dietetics. The intervention was conducted using an expository–dialogical approach, supported by standardized slide presentations (Microsoft PowerPoint, version 16.0, 2019).

Each NE session began with participant welcoming and topic contextualization, emphasizing its relevance to health promotion. The theoretical content was subsequently presented in a structured, progressive, and didactic manner, addressing key concepts related to healthy eating, appropriate food choices, and their associations with health and HIV. The slide presentations included concise text, illustrative images, and practical examples to facilitate comprehension and knowledge retention.

Participant engagement was actively encouraged throughout the sessions through guided questions, clarification of doubts, and exchange of experiences, fostering interaction and critical thinking. Each session lasted approximately 2 h. At the conclusion of each session, a summary of the main topics was provided to reinforce core messages, followed by an open discussion period for questions and final remarks.

The intervention covered the following topics: the act of eating and commensality; culinary skills; nutrition labeling; food groups (carbohydrates, proteins, lipids, dietary fiber, minerals, and vitamins); food hygiene; food preservation; healthy food choices; economically and regionally accessible foods; myths and facts about nutrition; nutrition in people living with HIV; dietary supplementation in people living with HIV; and the ten steps for adequate and healthy nutrition.

To promote adherence, participants received session reminders via a messaging application, including the date, location, and time of each session. Attendance was monitored using a sign-in sheet completed at the end of each session.

Following the completion of the NE sessions, participants in the intervention group received nutritional follow-up for 60 days through remote (virtual) counseling via a messaging application. This follow-up included individualized nutritional guidance, evaluation and prescription of laboratory tests, referrals to other health professionals when necessary, and clarification of questions arising during the follow-up period.

During both the intervention period and the subsequent 60-day follow-up, the control group did not receive any nutritional guidance or counseling in order to minimize contamination and bias. After completion of the intervention and the 60-day reassessment of both groups, the control group was offered the same NE sessions to ensure ethical parity.

### 2.8 Statistical analysis

All analyses mentioned below were performed using the open source software Jamovi^®^ (Version 2.3.18, Sydney, Australia), considering significance at *p* < 0.05. Data normality was tested using Shapiro-Wilk, Skewness, and Kurtosis (−1.96 / 1.96) tests and QQ-Line plotting. Thus, the data were expressed as mean and standard deviation. Subsequently, through a repeated measures ANOVA analysis, we performed comparisons between the different time points of the present study (baseline, 30 days to 60 days) considering the effect of the condition (Experimental vs. Control).

The assumption of homoscedasticity was tested using Levene's test, independence using the χ^2^ test, and sphericity using the Greenhouse-Geisser test. None of the assumptions were rejected. Specific differences were identified using Bonferroni's *post-hoc* test. The effect size between differences was determined by partial Eta-squared (η^2^p), considering the magnitude: small (< 0.01), medium (between 0.02 and 0.06) and large (>0.14) (25). Subsequently, results that showed statistically significant differences were expressed in graphs using GraphPad Prism 6.0 software.

## 3 Results

This study is an exploratory clinical trial. The primary outcome is the variable “Nutritional Knowledge,” and the secondary outcomes are the dependent variables related to: basal energy expenditure, body weight, body fat, and food intake.

All participants completed the assessments at the various time points (baseline, 30 days, and 60 days). Participants who did not complete the assessment stages were excluded from the study, and missing data were not included in the statistical analysis; the statistical analysis was performed only on complete records.

The sample consisted of 16 PLHIV, divided into two groups (experimental and control), with eight individuals per group. The data with the sociodemographic information of the research participants are shown in Table 1, indicating that most participants were male, single, had completed high school, had a monthly income of up to one base salary, and were between 28 and 59 years of age.

**Table 1:** Characteristics of the study population.

Variables	Total (*n* = 16)	Experimental (*n* = 08)	Control (*n* = 08)
Sex *n* (%)			
Male	13 (81.3)	6 (75.0)	7 (87.5)
Female	3 (18.8)	2 (25.0)	1 (12.5)
Age years μ (±)	47 (9.5)	48 (11.3)	46 (7.9)
Marital status *n* (%)			
Single	13 (81.3)	5 (62.5)	8 (100)
Married	1 (6.3)	1 (12.5)	00
Divorced	2 (12.5)	2 (25.0)	–
Education *n* (%)			
Complete high school	10 (62.5)	4 (50.0)	6 (75.0)
Complete college	6 (37.5)	4 (50.0)	2 (25.0)
Income *n* (%)			
Up to 1 BS	7 (43.8)	4 (50.0)	3 (37.5)
About 1 and 2 BS	6 (37.5)	2 (25.0)	4 (50.0)
15.6-7.5,-14.1242ptEqual or greater than 3 BS	3 (18.8)	2 (25.0)	1 (12.5)
Home *n* (%)			
Rented	3 (18.8)	2 (25.0)	1 (12.5)
Own home	10.(62.5)	4 (50.0)	6 (75.0)
Ceded	3 (18.8)	2 (25.0)	1 (12.5)
Physical conditioning *n* (%)			
Very active	6 (37.5)	4 (50.0)	2 (25.0)
Active	10 (62.5)	4 (50.0)	6 (75.0)
Infection time *n* (%)			
< 15 years	7 (43.8)	3 (37.5)	4 (50.0)
>15 years	9 (56.3)	5 (62.5)	4 (50.0)
ART time *n* (%)			
< 15 years	8 (50.0)	4 (50.0)	4 (50.0)
>15 years	8 (50.0)	4 (50.0)	4 (50.0)
ART *n*			
Lamivudina	13	6	7
Dolutegravir Tenofovir	15	7	8
Atazanavir	2	2	0
Ritonavir	1	1	0 1
Biovi	3	2	0 1
Darunavir	1 2	1 1	–

n: whole number. %: percentage. mean: μ; ±: standard deviation. ART, antiretroviral therapy; SB, base salary of R$1, 412.

Volunteers were recruited between February and April 2024. The follow-up period continued until August 2024. The clinical trial was interrupted due to the end of the postgraduate research period.

With regard to anthropometric data, most were classified as eutrophic according to BMI, with an average abdominal circumference above 90 centimeters.

**Figure 3:**
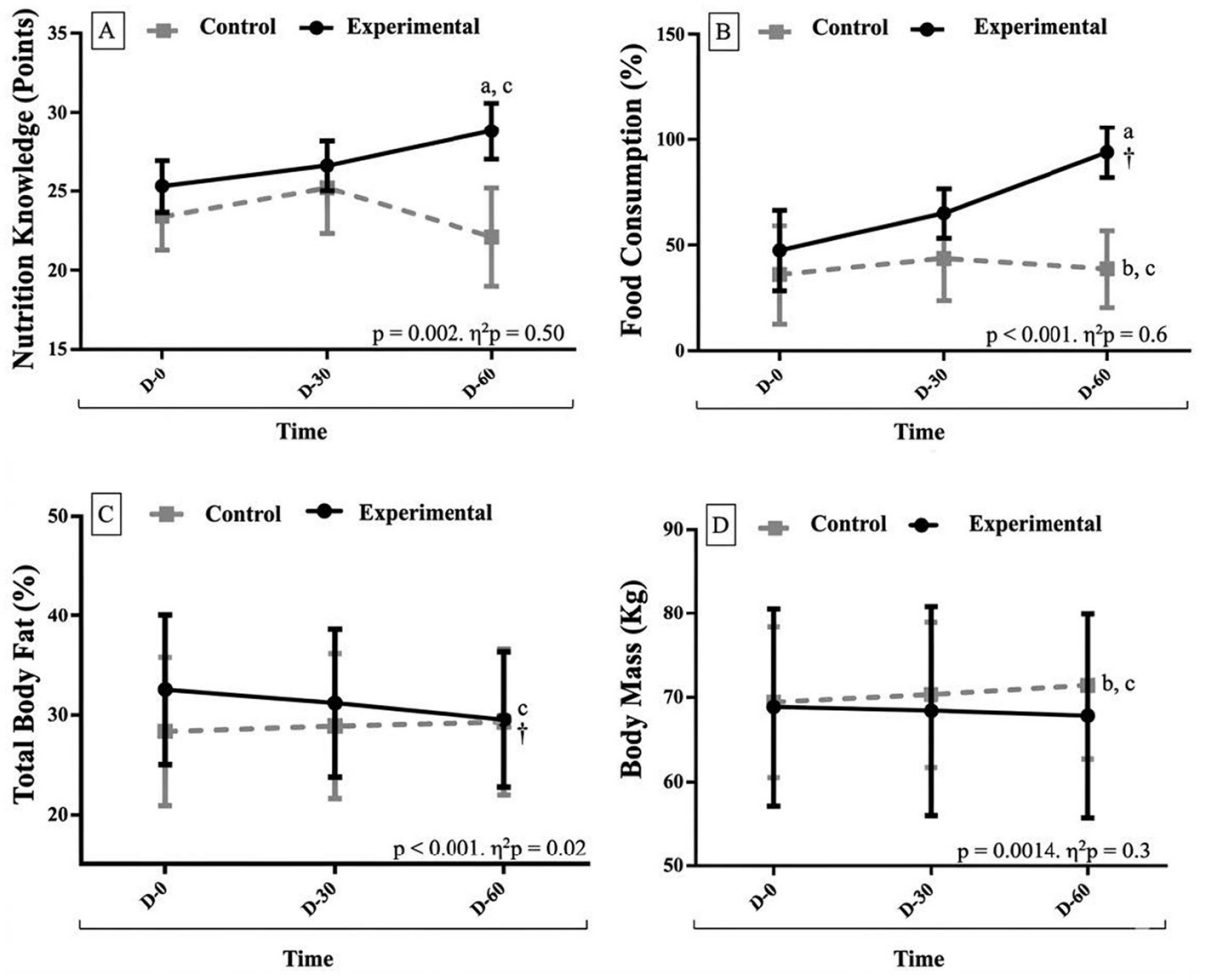
Comparisons between groups in relation to study variables. **(A)** nutritional knowledge. **(B)** Food Consumption. **(C)** total body fat. **(D)** Body Mass. D-0: Baseline (day zero). D-30: Analysis after thirty days. D-60: Final analysis after 60 days. %: Percentage. Kg: kilograms. η2p: partial eta-squared. Statistical analysis: Repeated measures ANOVA with Bonferroni *post-hoc*. c: Lower than the other time points. †: Higher than the other time points. a: Higher than the other conditions. b: Lower than the other conditions.

Detailed analyses are presented in Supplementary Table S1 of Supplementary Material 1, including model-derived *p*-values, Bonferroni-adjusted *p*-values, crude partial *R*^2^ values, and their corresponding 95% confidence intervals. The primary statistical findings are summarized below. The analyses revealed significant main effects of condition and time, as well as significant time × condition interactions for selected variables (Figure 3).

For nutritional knowledge, a significant main effect of condition was observed (*p* = 0.002), with higher scores in the experimental group compared with the control group at 60 days, as confirmed by the Bonferroni-adjusted *post hoc* test (*p* = 0.04). A significant time × condition interaction was also detected (*p* < 0.001), with differences between baseline in the control group and 60 days in the experimental group after Bonferroni correction (*p* = 0.005). Effect sizes were large for both condition (η^2^*p* = 0.498; 95% CI: 0.0947–0.6975) and the interaction (η^2^*p* = 0.458; 95% CI: 0.1270–0.6323) (Figure 3A).

For food consumption, a significant main effect of time was observed (*p* < 0.001), with higher values at 60 days compared with baseline, confirmed by Bonferroni-adjusted *post hoc* analysis (*p* < 0.001). A significant main effect of condition was also identified (*p* = 0.002), with higher values in the experimental group than in the control group at 60 days (Bonferroni-adjusted *p* = 0.04). In addition, a significant time × condition interaction was found (*p* < 0.001), with differences between baseline in the control group and 60 days in the experimental group after Bonferroni adjustment (*p* < 0.001). Effect sizes were large for time (η^2^*p* = 0.595; 95% CI: 0.2745–0.7285), condition (η^2^*p* = 0.501; 95% CI: 0.0975–0.6997), and the interaction (η^2^*p* = 0.565; 95% CI: 0.2385–0.7086) (Figure 3B).

It was found that 100% of the intervention group presented adequate food consumption markers, based on weekly consumption frequency for: raw vegetables (25%−75%) and cooked vegetables (12.5%−75%); fruits (25%−87.5%); beans (87.5%−100%); milk and dairy products (50%−87.5%); French fries, packaged potatoes, or fried snacks (37.5%−87.5%); hamburgers and sausages (50%−100%), crackers/savory biscuits or packaged snacks (62.5%−87.5%); sweet or filled cookies/biscuits, sweets, candies, and chocolates (50%−100%); and soft drinks (excluding diet drinks) (37.5%−100%).

Regarding body fat percentage (total fat %), a significant time × condition interaction was observed (*p* < 0.001), with differences identified by Bonferroni-adjusted *post hoc* analysis (*p* = 0.0411). Specifically, body fat percentage was lower at 60 days compared with other time points, and baseline values were lower in the control group than in the experimental group. The effect size was small (η^2^*p* = 0.024; 95% CI: 0.0000–0.2642) (Figure 3C).

For body mass (kg), a significant time × condition interaction was also observed (*p* = 0.0014), with differences confirmed by Bonferroni-adjusted *post hoc* analysis (*p* = 0.04). Body mass was higher at 60 days compared with other time points and was higher in the control group than in the experimental group at 60 days. The effect size was moderate (η^2^*p* = 0.302; 95% CI: 0.0145–0.5261) (Figure 3D). However, these findings should be interpreted with caution due to the short evaluation period and sample size.

Resting caloric expenditure, body fat in kilograms, BMI, MLG, and A/G ratio did not show significant differences in the tests (Table 2). However, a reduction in the A/G ratio was observed in the experimental group when comparing the data from the first assessment with that from 60 days (1.44–1.32), indicating a decrease in android fat, which reduces the risk of metabolic changes (23).

**Table 2:** Information on body composition and DXA analysis of participants.

Variables	Total (*n* = 16)	Experimental (*n* = 08)	Control (*n* = 08)
BMI *n* (%)			
Normal weight	10 (62.5)	4 (50.0)	6 (75.0)
Overweight	5 (31.3)	3 (37.5)	2 (25.0) 0
Obesity	1 (6.3)	1 (12.5)	
Circumference ABD in cm μ (±)			
Male	93.2 (7.9)	94.6 (8.8)	90.4 (7.9)
Female	90.8 (10.0)	89.3 (8.8)	98.1 (0)
Rate A/G *n* (%)			
>1	15 (93.7)	7 (87.5)	8 (100)
< 1	1 (2.3)	1 (12.5)	0
Total body fat in % *n* (%)			
Good	1 (6.3)	1 (12.5)	0 0 3 (37.5)
Below average	4 (25.0)	1 (12.5)	2 (25.0)
Average	1 (6.3)	1 (12.5)	3 (37.5)
Poor	6 (37.5)	4 (50.0)	
Very poor	4 (25.0)	1 (12.5)	
Total body fat in Kg μ (±)			
Male	20.1 (6.5)	19.9 (7.3)	20.3 (5.6)
Female	24.1 (5.5)	21.3 (3.5)	29.7 (0)
FFM in Kg μ (±)			
Male	50.5 (5.7)	50.1 (5.2)	50.9 (6.2)
Female	40.5 (5.4)	37.9 (4.3)	45.6 (0)

n: whole number. %: percentege. mean: μ. ±: standard deviation. A/G, android/gynoid; Kg, kilogram; BMI, body mass index; ABD, abdominall; FFM, fat-free mass; cm, centimeters.

BMI also decreased, mainly at 60 days, in the experimental group (26.2–25.9 kg/m^2^), reflecting a significant reduction in body mass. In the control group, however, BMI increased (24.3–25.1 kg/m^2^), indicating an increase in body mass in this group during this period.

## 4 Discussion

The results showed positive changes in the intervention group after 60 days of NE, where the nutritional knowledge of these individuals increased significantly and the food consumption marker showed improvement in weekly consumption frequency. The percentage of total body fat and body mass decreased significantly. In other words, when the knowledge gap about nutrition is filled through approaches focused on a specific population, in this case PLHIV, the study showed that NE can be beneficial in improving some nutritional parameters of these individuals.

### 4.1 Nutrition knowledge

In the intervention group, retention of nutrition/food knowledge passed on through NE was observed, as participants obtained higher scores when the questionnaire was reapplied 60 days after the intervention.

The questions that showed significant improvements in responses in the 60-day evaluation were those related to disease development, nutritional labeling, types of fat, sources of healthy fat, trans fat, sodium in food, healthy snacks, and diet and light foods.

These points were addressed during the NE with the following topics: food groups, nutritional labeling, healthy food choices, myths and truths about nutrition, and ten steps to proper and healthy eating.

A study conducted in Honduras, which also assessed nutrition knowledge after 60 days, with only two NE sessions during that period, found a significant improvement in nutrition knowledge among PLHIV (26).

Recent research shows that nutrition education or counseling can increase individual knowledge about healthy food choices in PLHIV (27–30). NE should be considered the first line of nutritional approach in HIV treatment, especially in developing countries with limited resources, as it is a cost-effective intervention (31).

### 4.2 Food consumption

Evidence suggests that NE is an essential component in the management of PLHIV, but few studies have been conducted in developing countries, including Brazil, to determine its effect on food consumption, to support or refute this evidence (10).

Therefore, this study found that 100% of the intervention group presented adequate consumption, based on weekly consumption of fresh or minimally processed foods (raw and cooked vegetables, fruits, beans, milk, and dairy products) five or more times per week, and processed and ultra-processed foods (hamburgers, sausages, cookies, and soda) less than or equal to twice a week.

A study conducted in Nigeria with PLHIV applied NE sessions for 2 months and found that fruits, vegetables, and legumes were significantly consumed in greater proportions in the intervention group, (32) as in the present study.

Since NE is built within the reality of the target audience, observing the gaps in knowledge about food/nutrition, understanding the social reality and regionality of the studied population, it has a positive impact on the frequency of consumption of fresh, minimally processed, processed, and ultra-processed foods.

However, a study conducted in Brazil found only adequate dietary fiber intake and a reduction in the percentage of lipid intake in the food consumption of 23 individuals with HIV after 12 months (one meeting every 2 months) of nutritional counseling (33). These findings contrast with the results observed in this study, suggesting that the methodology for constructing and applying the NE in the present study may have been more effective, both because of its focus on social conditions and because of the adaptations made for this specific population, considering the difficulties that were reported and the level of education.

As most participants had completed high school, the intervention proposal was designed exclusively for this audience. For NE interventions at lower school levels, adaptations are suggested, with information conveyed in a more playful way, seeking to bring it closer to their reality, in addition to using more simplified language.

### 4.3 Body composition

There is clear evidence that access to NE by individuals with HIV has potential factors that influence adequate nutrition, prolonging life and providing optimal nutritional status in PLHIV (34, 35).

NE plays a crucial role in promoting healthy eating practices, helping individuals develop confidence in their food choices and quantities to improve their nutritional status (36, 37). In addition, it is effective in modifying eating habits that influence chronic diseases, since a higher level of nutritional knowledge is positively and significantly associated with better dietary quality (38).

Even though the study volunteers were classified as physically active at the time of sample characterization, there was no standardized physical training program during the study period, which could have enhanced the results in relation to body composition.

It is possible to conjecture that, since NE is a consumption modifier by improving food intake, one can deduce a reduction in lipid consumption and an improvement in the dietary pattern of the study participants, which justifies the reduction in body mass and fat found (Table 3).

**Table 3:** Comparisons regarding the impact of food and nutrition education, considering the different groups (condition) and time points of the present study.

Variables	Conditions	Base line	30 days	60 days	Repeated measures ANOVA		
					p-Value (effect size by *η^2^p*)		
					Time	Condition	Interaction
Respiratory quotient	Experimental	0.86 ± 0.04	0.84 ± 0.04	0.85 ± 0.07	0.5 (0.05)	0.4 (0.05)	0.9 (0.01)
	Control	0.84 ± 0.05	0.83 ± 0.04	0.83 ± 0.04			
Resting energy expenditure (Kcal)	Experimental	1534 ± 217.4	1535 ± 234.6	1606 ± 273.1	0.6 (0.03)	0.5 (0.03)	0.05 (0.2)
	Control	1547 ± 237.7	1498 ± 243.1	1390 ± 279.6			
Fat (Kg)	Experimental	22.0 ± 7.3	21.3 ± 7.3	20.2 ± 6.9	0.4 (0.05)	0.9 (0.001)	0.6 (0.02)
	Control	22.9 ± 11.5	20.1 ± 6.7	21.5 ± 6.2			
FFM (Kg)	Experimental	46.9 ± 7.2	47.1 ± 7.9	48.1 ± 1.6	0.9 (0.07)	0.6 (0.04)	0.9 (0.002)
	Control	50.5 ± 5.2	50.4 ± 5.1	50.0 ± 7.1			
Rate A/G	Experimental	1.44 ± 0.5	1.36 ± 0.4	1.32 ± 0.3	0.07 (0.190)	0.4 (0.06)	0.149 (0.14)
	Control	1.24 ± 0.2	1.22 ± 0.2	1.23 ± 0.2			
BMI (Kg/m^2^)	Experimental	26.2 ± 3.4	26.1 ± 3.5	25.9 ± 3.2	0.5 (0.05)	0.4 (0.05)	0.03 (0.24)
	Control	24.3 ± 2.5	24.8 ± 3.0	25.1 ± 2.9			

Kcal, kilocalories; Kg, kilograms; m^2^, square meter; η^2^p, partial eta-squared; A/G, android/gynoid; BMI, body mass index; FFM, fat-free mass; Statistical analysis: repeated measures ANOVA with post-Hoc by Bonferroni.

In contrast, a study also conducted in Brazil with a similar population did not identify significant changes in body composition after nutritional intervention, even after stratification by sex, with three assessments (at baseline, 4 months, and 8 months after intervention) (39).

Studies indicate that it is difficult to significantly alter the body composition of PLHIV over different intervention periods, especially with regard to lean mass (40–42).

The low change in body composition in both genders may be related to the short intervention period, insufficient time to metabolize nutrients, lack of specific nutrient supplementation for PLHIV, side effects of antiretrovirals, low physical activity, and socioeconomic factors (Table 3).

### 4.4 Resting energy expenditure

With regard to energy expenditure at rest, QR and kcal/day were evaluated, but none of these variables showed significant changes (Table 3).

Resting energy expenditure is directly related to changes in FFM because this mass is mainly composed of metabolically active tissues that consume energy even at rest (43). Therefore, the absence of changes in resting energy expenditure is justified by the fact that the participants' FFM also did not show significant changes.

Medeiros et al. (2021) observed a reduction in resting metabolic rate in children and adolescents living with HIV, 4 and 6 months after dietary counseling with physical activity (39). Unlike the present study, which found no significant differences in this variable, Medeiros included physical activity and evaluated the effect of nutritional counseling over a longer period of time, which may have been the difference in identifying changes in resting energy expenditure.

### 4.5 Limitations and suggestions for future studies

Based on the understanding of NE as an intersectoral and transdisciplinary service, we suggest conducting multidisciplinary work with other health professionals, especially psychologists and physical education professionals, given that the HIV-positive population is also vulnerable in these areas.

A physical training program combined with NE would be interesting to investigate more significant bodily changes, observing trained and untrained individuals.

There was also a perceived difficulty in implementing more intensive interventions during a prolonged follow-up period, which limited the duration of the study due to the displacement of participants, since both the NE sessions and subsequent evaluations took place in person.

Regarding the assessment of food consumption, the authors make it clear that the consumption markers assess frequency rather than quantitative intake, so nutrient or energy intake was not estimated. A potential risk of reporting bias should be considered, as participants' dietary intake data were collected via self-report. To mitigate this bias, a structured questionnaire validated in the literature was employed.

For future studies, it is recommended that post-intervention assessments be conducted over a period longer than 60 days in order to evaluate the long-term effectiveness of NE, in addition to a quantitative assessment of food consumption.

### 4.6 Practical applicability

It is important to emphasize that education goes beyond simply imposing rules, so the transmission of knowledge alone can restrict the actions of NE. Eating is not just about ingesting nutrients essential for a good quality of life; it involves a complex set of meanings ranging from personal pleasure to sociocultural aspects in which the individual is inserted (44).

Thus, NE becomes more robust in nutritional care precisely because it considers factors beyond clinical ones. In PLHIV, this approach is necessary and brings short-term benefits due to the social norms and stigmas these people face. Therefore, nutritional assessment together with NE should be an integral part of HIV treatment programs.

In addition, NE can be effective when used in the context of public health, as it can be implemented in settings with limited financial and technological resources and conducted in large groups, which helps to expand nutritional care in situations where there is a shortage of nutritionists.

## 5 Conclusion

It was concluded that the nutrition education intervention was effective in improving nutritional parameters, increasing knowledge about nutrition, improving the frequency of consumption of foods with better nutritional density, and reducing the body mass and fat of participants after 60 days.

## Data Availability

The original contributions presented in the study are included in the article/Supplementary material, further inquiries can be directed to the corresponding author.
