# Supplementary material for: Nutrition education has a positive impact on nutritional knowledge, food consumption and body composition in PLHIV: a randomized clinical trial
Source: Front Public Health. 2026 May 20;14:1725861. doi: 10.3389/fpubh.2026.1725861 (PMC13230175; doi:10.3389/fpubh.2026.1725861)
Supplement: Supplementary file 1 [file Supplementary_File_1.pdf]

Table S1. Descriptive statistics.

	Group	Average	Confidence Interval 95% Lower limit	Confidence Interval 95% Upper limit	Standard-Deviation
**Knowledge T0**	**0**	23.44	21.67	25.20	2.112
	**1**	25.31	23.95	26.67	1.624
**Knowledge T1**	**0**	25.25	22.84	27.66	2.878
	**1**	26.63	25.31	27.94	1.575
**Knowledge T2**	**0**	22.06	19.44	24.68	3.133
	**1**	28.81	27.27	30.36	1.850
**Consumption T0**	**0**	36.25	16.80	55.70	23.261
	**1**	47.50	31.54	63.46	19.086
**Consumption T1**	**0**	43.75	27.07	60.43	19.955
	**1**	65.00	55.01	74.99	11.952
**Consumption T2**	**0**	38.75	23.64	53.86	18.077
	**1**	93.75	83.82	103.68	11.877
**Weight (Kg) T0**	**0**	69.51	62.04	76.99	8.943
	**1**	68.96	59.15	78.77	11.734
**Weight T1 (Kg)**	**0**	70.42	63.23	77.62	8.607
	**1**	68.54	58.16	78.92	12.414

**Weight T2 (Kg)**	**0**	70.81	63.64	77.98	8.574
	**1**	67.90	57.80	77.99	12.077
**% Total Fat T0**	**0**	28.36	22.15	34.57	7.428
	**1**	32.54	26.27	38.81	7.498
**% Total Fat T1**	**0**	28.89	22.82	34.95	7.252
	**1**	31.19	25.00	37.38	7.406
**%Total Fat T2**	**0**	29.31	23.18	35.44	7.330
	**1**	29.54	23.88	35.20	6.771
**Kcal/d T0**	**0**	1547.25	1348.50	1746.00	237.731
	**1**	1533.88	1352.14	1715.61	217.376
**Kcal/d T1**	**0**	1497.50	1294.29	1700.71	243.064
	**1**	1534.88	1338.73	1731.02	234.618
**Kcal/d T2**	**0**	1389.88	1156.14	1623.61	279.581
	**1**	1606.25	1377.90	1834.60	273.138
**Fat (Kg) T0**	**0**	22.95	13.33	32.57	11.512
	**1**	22.03	15.95	28.10	7.269
**Fat (Kg) T1**	**0**	20.05	14.41	25.69	6.744
	**1**	21.27	15.20	27.35	7.264
**Fat (Kg) T2**	**0**	21.50	16.34	26.66	6.172
	**1**	20.24	14.43	26.05	6.950

**FMM (Kg) T0**	**0**	50.49	46.13	54.84	5.211
	**1**	46.89	40.85	52.92	7.217
**FMM (Kg) T1**	**0**	50.35	46.08	54.62	5.109
	**1**	47.14	40.47	53.80	7.975
**FMM (Kg) T2**	**0**	50.23	45.22	55.24	5.994
	**1**	47.09	40.73	53.45	7.606
**Rate A/G T0**	**0**	1.24	1.06	1.42	0.218
	**1**	1.44	1.06	1.82	0.454
**Rate A/G T1**	**0**	1.22	1.06	1.38	0.196
	**1**	1.36	1.06	1.65	0.355
**Rate A/G T2**	**0**	1.23	1.09	1.38	0.172
	**1**	1.32	1.05	1.59	0.326
**BMI T0**	**0**	24.35	22.23	26.47	2.536
	**1**	26.23	23.37	29.08	3.410
**BMI T1**	**0**	24.76	22.30	27.23	2.947
	**1**	26.06	23.13	29.00	3.509
**BMI T2**	**0**	25.07	22.69	27.46	2.853
	**1**	25.91	23.23	28.60	3.214

T0: baseline. T1: 30 days. T2: 60 days. Group 0: control. Group 1: experimental. Kcal/d: kilocalories/day. Kg: kilograms. A/G: android/gynoid. BMI: body mass index. FFM: fat-free mass.
